# Efficacy of Myofascial Release Therapy and Related Soft‐Tissue Techniques for Pain in Temporomandibular Disorders: A Systematic Review of Randomized Controlled Trials

**DOI:** 10.1111/joor.70271

**Published:** 2026-07-15

**Authors:** Martina Ferrillo, Dario Calafiore, Alfredo De Rosa, Ambra Sedran, Umile Giuseppe Longo, Michele Vecchio, Antonio Ammendolia, Nicola Scotti, Amerigo Giudice, Alessandro de Sire

**Affiliations:** ^1^ Dentistry Unit, Department of Health Sciences University of Catanzaro “Magna Graecia” Catanzaro Italy; ^2^ Physical Medicine and Rehabilitation Unit, Department of Neuroscience ASST Carlo Poma Mantova Italy; ^3^ Department of Life Science, Health and Health Professions Link Campus University Rome Italy; ^4^ Dental School, Department of Surgical Sciences University of Turin Turin Italy; ^5^ Fondazione Policlinico Universitario Campus Bio‐Medico Roma Italy; ^6^ Research Unit of Orthopaedic and Trauma Surgery, Department of Medicine and Surgery Università Campus Bio‐Medico di Roma Roma Italy; ^7^ Department of Biomedical and Biotechnological Sciences University of Catania Catania Italy; ^8^ Physical Medicine and Rehabilitation Unit, AOU Policlinico G. Rodolico University of Catania Catania Italy; ^9^ Research Center on Musculoskeletal Health, MusculoSkeletalHealth@UMG University of Catanzaro “Magna Graecia” Catanzaro Italy; ^10^ Physical Medicine and Rehabilitation Unit, Department of Medical and Surgical Sciences University of Catanzaro “Magna Graecia” Catanzaro Italy

**Keywords:** myofascial release, pain, physiotherapy, rehabilitation, temporomandibular diseases

## Abstract

**Objective:**

This systematic review aimed to evaluate the effectiveness of myofascial release therapy (MFR) on pain in patients affected by myogenous temporomandibular disorders (TMD).

**Methods:**

PubMed, Scopus, and Web of Science were searched from inception until 31 January 2026, to identify RCTs including patients affected by TMD as participants, MFR therapy as intervention, and pain intensity as outcome. The risk of bias of studies was assessed with the Version 2 of the Cochrane risk‐of‐bias tool for randomized trials (RoB 2). The Grading of Recommendations Assessment, Development and Evaluation (GRADE) approach was used to assess the certainty of evidence.

**Results:**

Out of 857 articles, 7 studies were included. All subjects had a diagnosis of TMD performed according to the RDC/TMD or DC/TMD. The rehabilitation period ranged from 10 days to 5 weeks. Treatment frequency ranged from a minimum of one session per week to a maximum of 3 sessions per week. Medium/long‐term follow‐up was performed in four studies. The Cochrane RoB 2 tool showed that zero studies had a low risk of bias, six had some concerns, and one had a high risk of bias. The GRADE assessment showed a low certain of evidence for pain reduction and mouth function, and a very low certain of evidence in improving QoL.

**Conclusions:**

MFR seemed to reduce pain and improve mouth function in patients with myogenous TMD. However, when benchmarked against established clinical thresholds, clinically meaningful efficacy (surpassing the 15–22 mm MCID) is only supported by a subset of the included trials. In this context, MFR could be considered as part of a multidisciplinary approach for patients with TMD. More rigorous isolated‐MFR trials are needed to better characterize the efficacy of MFR in patients affected by myogenous TMD.

## Graphical abstract

This systematic review evaluated the effectiveness of myofascial release therapy (MFR) for pain management in patients with myogenous temporomandibular disorders (TMD). Seven randomized controlled trials involving patients diagnosed according to RDC/TMD or DC/TMD criteria were included. MFR was associated with reduced pain intensity and improved mouth function, although the certainty of evidence was low according to GRADE. Only a subset of studies achieved clinically meaningful pain reduction beyond the minimal clinically important difference. Current evidence supports considering MFR as part of a multidisciplinary rehabilitation approach for myogenous TMD, while highlighting the need for larger, high‐quality randomized controlled trials evaluating isolated MFR interventions.

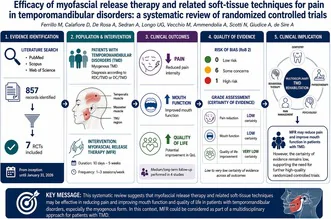

## 1 Introduction

Temporomandibular disorders (TMD) are a collection of musculoskeletal disorders in the temporomandibular region [1]. TMD aetiology is multifactorial, comprising biomechanical dysfunctions, psychosocial stressors, neuromuscular imbalances, hormonal influences, and anatomical irregularities [2]. Moreover, several risk factors that include genetics, muscle or joint disturbances, anatomical factors, trauma, and systemic conditions such as rheumatic diseases, are associated [3, 4, 5]. These conditions are characterized by pain and dysfunction of masticatory muscles, the temporomandibular joint (TMJ), and associated structures [6]. TMD symptoms include joint sounds, pain, and restrictions in mandibular movements, and cause difficulty in performing basic daily activities such as chewing, swallowing, and talking [7].

The Diagnostic Criteria for Temporomandibular Disorders (DC/TMD) Axis I classifies TMD into pain disorders including myalgia, arthralgia, and headache attributed to TMD and joint disorders including disc displacement conditions, degenerative joint diseases, and subluxation [8, 9]. Myogenous TMD can be further categorized into: local myalgia, if the pain is localized during palpation; myofascial pain, if the pain spreads within the palpated muscular territory; and myofascial pain with referral, if the pain spreads beyond the boundary of the masticatory muscles [8].

TMD have been reported to be a significant public health problem [10, 11, 12], and a recent meta‐analysis suggested that female gender was associated with a higher risk of developing TMD [13].

Conservative management is the preferred first‐line approach for most TMD cases, particularly those of myofascial [14]. One of the most widely adopted conservative therapies is the use of physiotherapy, instrumental physical therapy, and manual therapy, including myofascial release therapy (MFR) that aim to alleviate muscle hyperactivity, fascial pain, and reduce joint loading [15]. Based on current understanding of the muscle structure and function, myofascial physiotherapy techniques were implemented in a stepwise manner, with incremental increases in intensity throughout the intervention period [16]. MFR is a widely employed manual therapy treatment that involves guided low load and long‐duration mechanical forces to manipulate the myofascial complex, intended to restore optimal length, decrease pain, and improve function [17]. The origin of myofascial release can be traced back to the 1940s, but the term myofascial release was not proposed until 1981 [18]. In recent years, MFR has been applied to the rehabilitation treatment of musculoskeletal injuries to provide immediate relief of pain and tissue tenderness in several musculoskeletal diseases such as neck pain, low back pain, scapulohumeral periarthritis, and functional ankle instability, and the clinical application and related experiments of MFR show an increasing trend [19, 20, 21]. MFR generally involves slow, sustained pressure (120–300 s) applied to restricted fascial layers either directly (direct MFR technique) or indirectly (indirect MFR technique) [22]. Direct MFR technique is thought to work directly over the restricted fascia using knuckles or elbow or other tools to slowly sink into the fascia, and the pressure applied is a few kilograms of force to contact the restricted fascia, apply tension, or stretch the fascia [23]. Indirect MFR involves a gentle stretch guided along the path of least resistance until free movement is achieved [24]. The pressure applied is a few grams of force, and the hands tend to follow the direction of fascial restrictions, hold the stretch, and allow the fascia to loosen itself [25]. The rationale for these techniques can be traced to various studies that investigated plastic, viscoelastic, and piezoelectric properties of connective tissue [26]. Several authors have suggested that connective tissue could become tighter/denser in overuse syndromes, or after traumatic injuries, but it is unclear if this is due to an alteration of collagen fibre composition, of fibroblasts, or of ground substance [27, 28]. MFR practitioners claim to be clinically efficacious in providing immediate pain relief and to improve physiologic functions that have been altered by somatic dysfunctions [29, 30]. In addition, MFR promotes soft tissue release and extension, enhances local blood circulation, and restores the range of motion of restricted joints, thereby improving muscle pain, stiffness, or excessive fatigue to a certain extent [17, 31]. MFR directs force to fascial fibroblasts, as well as indirect strains applied to nerves, blood vessels, the lymphatic system, and muscles. Laboratory experiments suggest that fibroblasts, the primary cell type of the fascia, adapt specifically to mechanical loading in manners dependent upon the strain magnitude, duration, and frequency [32]. MFR reduces the tension of a muscle or even an entire muscle group, as it inhibits the motoneuron field of a given muscle and thus leads to reflex relaxation [33]. The reason for this is the activation of the Golgi tendon organs during contraction [34]. There are two MFR targets—short‐term and long‐term. The immediate goal is primarily to alleviate pain and other effects of static muscle overload and to reduce muscle and connective tissue irritation [35]. On the other hand, the long‐term goal is to restore the expected length and flexibility of contracted muscles, regain normal joint range of motion, decreasing potential overload of synovial tissue [36].

To date, findings emphasize the effectiveness of MFR in the treatment of TMD, achieving both muscle relaxation, improved tissue blood supply and joint range of motion, and reduced pain [37]. However, there is a lack of systematic reviews of controlled randomized trials (RCTs) that properly investigated the efficacy of MFR in reducing pain in patients affected by TMD. Therefore, this review focused on MFR and related soft‐tissue techniques for pain in TMD, including direct MFR, instrument‐assisted MFR (IASTM), soft tissue mobilization, and multimodal therapies in which MFR is one component. Given the heterogeneity of interventions, we present technique‐specific results where possible and refrain from attributing effects to MFR as intervention.

## 2 Materials and Methods

This systematic review was registered in PROSPERO under registration number CRD420251161805. The protocol for this review was registered in PROSPERO on 5 October 2025. The review was conducted and reported in accordance with the PRISMA 2020 statement. During the conduct of the review, several amendments to the registered protocol were made. These amendments concerned the population, diagnostic criteria, information sources, eligible comparators, additional search methods, search dates, and review team composition, in accordance with PRISMA 2020 item 24 [38, 39]. Briefly, although the original protocol included all DC/TMD diagnostic groups, the deviations from the registered protocol include: (1) expansion of included study designs to randomized trials only (if applicable), (2) explicit pre‐specification of intervention‐type subgroups (direct MFR, instrument‐assisted MFR, soft‐tissue mobilization, multimodal therapies containing MFR), (3) updated risk‐of‐bias assessment with RoB 2. Final review focused on patients with myogenous TMD because the intervention under investigation, namely myofascial release, primarily targets muscular and fascial components. Studies using the RDC/TMD were also considered eligible when the diagnostic categories were compatible with myogenous TMD. In the revised version, searches were performed from inception to 31 January 2026 in PubMed/MEDLINE, Scopus, and Web of Science. Additional searches included screening the reference lists of relevant systematic reviews and included studies, forward citation searching, and searches of trial registries. Where necessary, study authors were contacted for clarification or additional information.

### 2.1 Eligibility Criteria

Randomized controlled trials (RCTs) assessed for eligibility according to the following participants, intervention, comparison, and outcomes (PICOS) model:

1. Participants: adult patients with a diagnosis of TMD according to the DC/TMD [8] or to the Research Diagnostic Criteria for Temporomandibular Disorders (RDC/TMD) [40].
2. Intervention: myofascial release therapy;
3. Comparison: Comparison consisted of placebo, sham treatments, behavioural therapy, or conservative approaches (e.g., physical therapy, laser therapy, occlusal splints, or any other type of conservative treatment);
4. Outcome: pain intensity, using visual analogue scale (VAS) or numerical rating scale (NRS); secondary outcome if available: mouth function, Quality of Life (QoL), adverse effect.
5. Study design: RCTs

Exclusion criteria were (1) children or adolescents during growth; (2) patients with a history of TMJ trauma; (3) patients suffering from any rheumatic diseases or inflammatory disorders (e.g., rheumatoid arthritis); (4) patients with congenital abnormalities or neoplastic conditions in the TMJ region; (5) cross‐over study design; (6) studies involving animals; (7) studies written in a language different from English; and (8) non‐peer‐reviewed studies (i.e., posters and conference abstracts).

### 2.2 Search Strategy

During the initial phase of the study, a systematic literature search was conducted using specific keywords within the major scientific databases, according to each specific thesaurus, following the strategy described by Table 1.

**TABLE 1:** Search strategy.

PubMed
(‘temporomandibular joint dysfunction syndrome’[MeSH] OR ‘temporomandibular joint disorder’ OR ‘temporomandibular disorder’ OR ‘temporomandibular dysfunction’ OR ‘TMD’ OR ‘temporomandibular joint’ OR ‘TMJD’ OR ‘craniomandibular disorders’[MeSH] OR ‘craniomandibular dysfunction’ OR ‘temporomandibular joint dysfunction’) AND (‘myofascial release therapy’[MeSH] OR ‘myofascial release’ OR ‘myofascial treatment’ OR ‘myofascial therapy’ OR ‘manual therapy’ OR “’myofascial relaxation’)
Scopus
TITLE‐ABS‐KEY ((‘temporomandibular joint dysfunction syndrome’ OR ‘temporomandibular joint disorder’ OR ‘temporomandibular disorder’ OR ‘temporomandibular dysfunction’ OR ‘TMD’ OR ‘temporomandibular joint’ OR ‘TMJD’ OR ‘craniomandibular disorders’ OR ‘craniomandibular dysfunction’ OR ‘temporomandibular joint dysfunction’) AND (‘myofascial release therapy’ OR ‘myofascial release’ OR ‘myofascial treatment’ OR ‘myofascial therapy’ OR ‘manual therapy’ OR ‘myofascial relaxation’))
Web of Science
((‘temporomandibular joint dysfunction syndrome’ OR ‘temporomandibular joint disorder’ OR ‘temporomandibular disorder’ OR ‘temporomandibular dysfunction’ OR ‘TMD’ OR ‘temporomandibular joint’ OR ‘TMJD’ OR ‘craniomandibular disorders’ OR ‘craniomandibular dysfunction’ OR ‘temporomandibular joint dysfunction’) AND (‘myofascial release therapy’ OR ‘myofascial release’ OR ‘myofascial treatment’ OR ‘myofascial therapy’ OR ‘manual therapy’ OR ‘myofascial relaxation’))

### 2.3 Data Extraction

Two reviewers independently extracted data from included studies using a customized data extraction on a Microsoft Excel sheet. In case of disagreement, the consensus was achieved through a third reviewer. Following data were extracted: (1) First author; (2) Publication year; (3) Journal; (4) demographic characteristics of study participants; (5) TMD diagnosis; (6) rehabilitative approach as intervention; (7) Type of rehabilitative approach as comparison (placebo, sham treatments, or the approaches considered as interventions), if available; (8) Time points; (9) Main findings. Baseline pain (primary outcome) and end‐of‐treatment pain were extracted. We reported mean ± SD and median [IQR] according to statistical analysis of the studies. A single standardized table (Table 2) lists for each trial was performed. Number, sex and age of patients, intervention arm, baseline pain, end‐of‐treatment pain, and brief description of the results. To evaluate the clinical relevance of the interventions, an a priori Minimum Clinically Important Difference (MCID) benchmark was established for the primary outcome of pain intensity (measured via VAS or NRS). Based on validated, TMD‐specific clinimetric literature, we utilized an MCID threshold range of 15–22 mm on a 100 mm scale (or 1.5–2.2 points on a 10‐point scale). This range reflects the thresholds for clinical meaningfulness and substantial clinical benefit established [41]. Throughout this review, effect estimates are evaluated not only for statistical significance but also benchmarked against this MCID to determine clinically meaningful improvement. Moreover, a narrative synthesis of the findings was conducted.

**TABLE 2:** Main characteristics of the studies included in the present systematic review.

Authors, publication year, journal	Population (M/F)	Age	TMD diagnosis	Intervention	Comparison	Outcomes	Time‐points	Main findings in terms of primary outcome
Brochado et al. 2018, Braz Oral Res	Total: *n* = 51 5 M/46 F MT group: 3 M/13 F PBM group: 2 M/16 F MT + PBM group: 3 M/14 F	Total: 44.5 ± 17.1 years MT group: *41.2 ± 20.3* years PBM group: *45.7 ± 15.7* years Control group: *46.5 ± 14.4* years	According to the RDC/TMD	MT group: patients underwent 3 sessions/week for 21‐min sessions for 4 consecutive weeks.	MT + PBM group: Patients underwent PBM and MT, 3 times/week for 4 consecutive weeks. PBM group: patients underwent professional session with a continuous wave of GaAlAs diode laser, with a wavelength of 808 Nm, 12 times (3 times a week for 4 consecutive weeks).	Pain assessed with a visual analogue scale (VAS), pain‐free maximum mouth opening, maximum mouth opening, maximum assisted mouth opening, right and left lateral movement, and protrusion., RDC/TMD Axis II assessments, maximum mouth opening, and BAI.	Data were collected at T0 (baseline), at T1 (7 days), T2 (14 days), T3 (21 days), T4 (28 days), T5 (60 days) T6 (90 days).	Significant pain and improvement in jaw movements were shown at both T1 and T2 (*p* < 0.001). All three groups experienced a significant reduction in pain by D14 (*p* < 0.001). The change in mean VAS scores did not differ significantly between groups during evaluation time. In D90, all groups maintained a stable mean similar to D28 (*p* < 0.05). The Axis II evaluation revealed a not significant improvement of the intensity of chronic pain at follow‐up in all treatment groups (*p* > 0.05). However, a significant reduction of depression and BAI was shown in all groups (*p* ≤ 0.05).
Leite et al., 2020, J Manipulative Physiol Ther	Total: *n* = 34 0 M/34 F DF group: 0 M/17 F Control group: 0 M/17 F	Total: 44.5 ± 17.1 years DF group: *27.1 ± 7.25* years Control group: *23.4 ± 5.62* years	According to the RDC/TMD	DF group: patients underwent two sessions/week for 4 weeks (8 sessions) of real DF. The time needed for each DF session was about 10 min.	Control group: sham therapy	Pain assessed with a visual analogue scale (VAS), maximum mouth opening, PPTs at the TMJ, temporalis, and masseter muscles.	Data were collected at T0 (baseline), at T1 (4 weeks).	Both groups showed decreased pain after 4 weeks, but the DF showed lower results compared with sham, with significant differences between groups at the 4‐week assessment (*p* < 0.01). The DF had lower PPT whereas the controls had higher PPT, both compared with each baseline results. The time‐by‐group interaction showed a significant difference for the DF over time (*p* = 0.017) for mouth opening.
Serrano‐Hernanz et al., 2023, J Oral Rehabil	Total: *n* = 72 12 M/60 F PRT group: 8 M/29 F Control group: 4 M/31 F	PRT group: 46.9 ± 14.0 years Control group: 36.6 ± 13.2 years	According to the DC/TMD	PRT group: patients underwent one session/week for 45‐min for 5 weeks of pressure release technique to the trigger points of both sides of the masticatory and cervical muscles.	Control group: sham therapy	Pain assessed with a visual analogue scale (VAS), PPTs at the temporalis, masseter, sternocleidomastoid, and trapezius muscles, maximum mouth opening, NDI, PCS, TSK‐11, STAI, and ST‐DEP.	Data were collected at T0 (baseline), at T1 (end of intervention), T2 (3 months post intervention).	Findings showed a significant reduction of VAS pain between groups (*p* < 0.001). Also, there were significant effects of time in secondary outcomes (*p* < 0.001), and there were also interactions between time and group (*p* < 0.002) with better effects in the PRT group.
Romeo et al., 2024, J Oral Rehabil	Total: *n* = 62 13 M/49 F MPT group: 5 M/24 F Control group: 8 M/25 F	MPT group: 38.48 ± 17.75 years Control group: 31.88 ± 9.10 years	According to the DC/TMD	MPT group: patients underwent MPT, occlusal splint therapy, and education. MPT was composed by 10 sessions of musculoskeletal physiotherapy, lasting 45 min each and delivered in 3 months.	Control group: occlusal splint therapy and education.	Pain assessed with a visual analogue scale at rest (VAS rest), at maximum oral opening (VAS open), and during chewing (VAS chew), maximum mouth opening.	Data were collected at T0 (baseline), at T1 (end of intervention), T2 (3 months post intervention).	Between‐group analysis showed significant reduction of VAS rest (*p* = 0.04), VAS open (*p* < 0.01), VAS chew (*p* = 0.01), and maximum mouth opening (*p* = 0.04) in favour of the MPT group.
Espejo‐Antúnez et al., 2024, J Appl Oral Sci	Total: *n* = 46 0 M/46 F ES + MT group: 0 M/25 F MT group: 0 M/21 F	ES + MT group: 23.92 ± 7.14 years MT group: 26.24 ± 9.42 years	According to the DC/TMD	MT group: patients underwent soft tissue mobilization and release techniques.	ES + MT group: patients underwent soft tissue mobilization and release techniques plus dynamic cervical electrical stimulation (electro‐massage) two sessions/week for two weeks. Each MT session lasted for approximately 25 min.	Pain assessed with a visual analogue scale (VAS), PPTs at the masseter and upper trapezius muscles, maximum mouth opening.	Data were collected at T0 (baseline), at T1 (end of intervention), T2 (4 weeks post intervention).	Significant changes regarding pain intensity (*p* < 0.00), pressure pain sensitivity and maximum mouth opening (*p* < 0.001) were shown in favour of the ES + MT group. Similar results were reported for active cervical range‐of‐movement in all directions (*p* < 0.001), except rotation (*p* ≥ 0.05).
Ferreira et al., 2024, J Bodyw Mov Ther	Total: *n* = 60 24 M/36 F DN group: 4 M/16 F MT group: 5 M/15 F Control group: 15 M/15 F	Total: 44.5 ± 17.1 years DN group: *31.3 ± 6.2* years MT group: *30.6 ± 5.9* years Control group: *29.6 ± 6.9* years	According to the DC/TMD	MT group: patients underwent manual therapy 30‐min session/week for 4 weeks.	Control group: patients underwent cognitive‐behavioural therapy 30‐min session/week for 4 weeks. DN group: patients underwent dry needling 30‐min session/week for 4 weeks.	Pain assessed with a visual analogue scale (VAS), maximum mouth opening, right and left lateral movement, and protrusion without pain.	Data were collected at T0 (baseline), at T1 (end of intervention), T2 (30 days post intervention).	Results showed a decrease in pain scores over time in the DN and MT groups (*p* < 0.001). The maximum mouth opening increased in the DN and MT groups (*p* = 0.005), while protrusion increased only in the DN group (*p* = 0.007).
Gurudut et al., 2025, Int J Ther Massage Bodywork	Total: *n* = 34 17 M/17 F MT group: 7 M/10 F Control group: 10 M/7 F	MT group: *25.41 ± 8.39* years Control group: *34.41 ± 10.83* years	According to the RDC/TMD	MT group: patients underwent soft tissue manipulation including massage and myofascial release for facial muscles for 5 sessions on alternate days (each session lasting 20–30 min).	Control group: patients underwent drug therapy with chlorzoxazone 250 mg and aceclofenac 100 mg for 7 days twice daily.	Pain assessed with a numeric pain ratingsScale (NPRS), Chronic Graded Pain Version 2.0 (CGPV 2.0), maximum mouth opening (MMO), and TMJ Disability Index (TDI) scale.	Data were collected at T0 (baseline) and at T1 (end of intervention).	Both groups showed a statistically significant difference in pain (*p* = 0.001), MMO (*p* = 0.001), CGPV 2.0 (*p* = 0.001), and TDI (*p* = 0.001). However, no significant inter‐group difference were reported for NPRS (*p* = 0.066), whereas significant difference in MMO (*p* = 0.001), CGPV 2.0 (*p* = 0.001), and TDI (*p* = 0.001) were shown between both the groups.

*Note:* Values are presented as mean ± standard deviation and maximum‐minimum (range).

Abbreviations: BAI, beck anxiety inventory; DC/TMD, diagnostic criteria for temporomandibular disorders; DF, diacutaneous fibrolysis; DN, dry needling; ES, dynamic cervical electrical stimulation; F, female; IMT, intraoral myofascial therapy; IMTESC, intraoral myofascial therapy, education, and self‐care group; M, male; MPT, musculoskeletal physiotherapy; MT, manual therapy; N/A, not applicable; NDI, neck disability Index; PBM, photobiomodulation therapy; PCS, pain catastrophizing scale; PPT, pressure pain threshold; PRT, pressure release technique; RDC/TMD, research diagnostic criteria for temporomandibular disorders; STAI, State–Trait anxiety index; ST‐DEP, State–Trait Depression Index; TMD, temporomandibular disorders; TSK‐11, Tampa Scale for Kinesiophobia; VAS, visual analogue scale.

### 2.4 Quality Assessment

Two independent reviewers (M.F. and D.C.) conducted the risk of bias assessment. Disagreements between the two assessors were resolved through discussion or by consulting a third reviewer (A.d.S.). To quantify the level of agreement between the two independent reviewers, a weighted Cohen's kappa (K) statistic was calculated, in accordance with the PRISMA 2020 guidelines [42]. Any disagreement was discussed until a consensus was reached with a third reviewer. We used the Grading of Recommendations Assessment, Development and Evaluation (GRADE) approach to assess the certainty of evidence for direct, indirect, and network estimates for all outcomes. With this approach, the certainty of direct evidence from randomized trials starts as high but may be rated down for risk of bias, indirectness, imprecision, inconsistency, or small study effects to moderate, low, or very low [43].

## 3 Results

### 3.1 Study Characteristics

**FIGURE 1:**
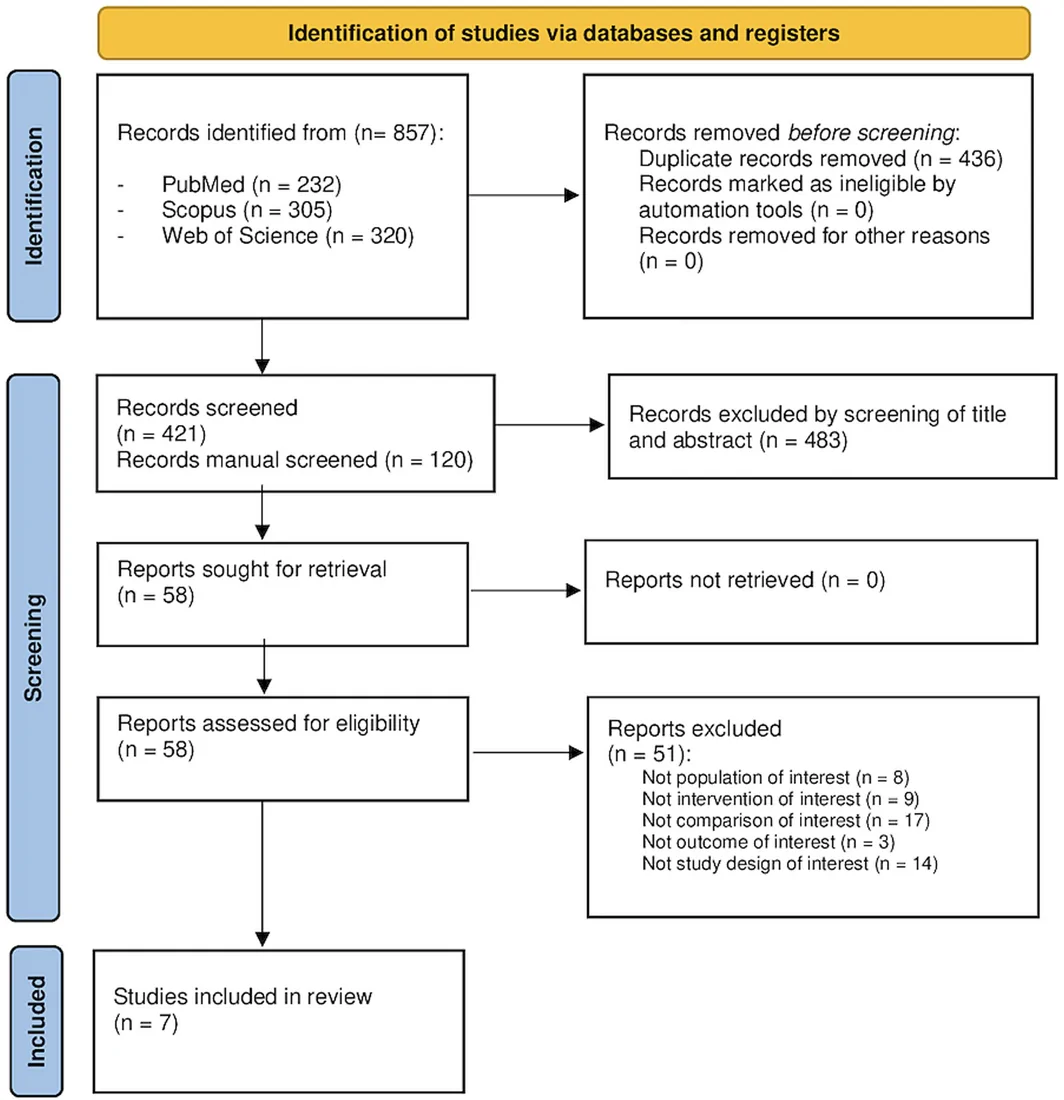
2020 PRISMA flow diagram.

The electronic search generated a total of 857 articles. After removing duplicates, 421 records were screened for title and abstract. Out of these, 58 articles were assessed and 51 were excluded, thus 7 [44, 45, 46, 47, 48, 49, 50] studies were included in the present systematic review, as reported by the 2020 PRISMA flow diagram (see Figure 1). The descriptive characteristics and main findings of the included studies are presented in Table 2. Across the seven included trials, participants presented with various classifications of TMD. While the majority of the studies focused predominantly on myogenous TMD, trials enrolling mixed populations (myogenous and arthrogenic), such as Brochado et al. (2018) [44], were included in accordance with our predefined eligibility criteria (DC/TMD Groups I, II, and III). The publication year of the studies ranged between 2018 and 2025. The number of participants per study ranged from 34 to 72, with a total of 359 study participants, of which 71 were males and 288 were females. The average ages of the study cohorts ranged from 23 to 46 years. All subjects had a diagnosis of TMD performed according to the RDC/TMD [44, 45, 46] or DC/TMD [47, 48, 49, 50]. The rehabilitation period ranged from 10 days [46] to 5 weeks [47]. Treatment frequency ranged from a minimum of one session per week [47, 48, 50] to a maximum of 3 sessions per week [44, 46]. Each session lasted from a minimum of 10 min [43] to a maximum of 45 min [47, 48]. All studies included an initial pain assessment at T0, but reassessment timing was not homogeneous. Pain reassessment at the end of treatment was conducted in five studies, [46, 47, 48, 49, 50] whereas pain reassessment 4 weeks after intervention was performed in one study [43]. Follow‐up assessment timelines varied across the included studies. Medium/long‐term follow‐up was performed in four studies: two studies [47, 48] assessed the outcomes 3 months after intervention, one study [50] 30 days post intervention, one study [44] at 60 and 90 days from baseline, which corresponds to approximately 4 and 8 weeks post‐treatment. Conversely, some studies only assessed outcomes at the end of the treatment period without follow‐up [45, 46].

T Brochado et al. [44] included a total of 51 participants with TMD. The participants were divided into three groups: 16 in the experimental group treated with manual therapy (MT group), 18 in the group treated with photobiomodulation (PBM) therapy (PBM group), and 17 in the group treated with both techniques in each session (MT + PBM group). All treatments were performed 3 times/week for 4 consecutive weeks. MT involved a 21‐min session performed with circular movements of the finger on the face, with progressive pressure adapted to the tissue and the patient's sensitivity, also extended to the intraoral region of the masseter. PBM was applied with a continuous GaAlAs diode laser (808 nm) on 12 specific points. Pain assessment was performed weekly during the treatment period (days 7, 14, 21, and 28) and at the 4‐week (day 60) and 8‐week (day 90) follow‐ups after the conclusion of therapy using a VAS scale. All groups showed a reduction in pain by day 14 compared to T0 (at T0: MT: 4.4 [2.46–6.31]; PBM: 4.1 [2.91–5.38]; MT + PBM: 5.2 [3.75–6.6] and at T14: MT: 1.7 [0.71–2.68], PBM: 1.9 [1.10–2.76]; MT + PBM: 2.4 [1.72–3.13], all *p* < 0.001). Throughout the entire evaluation period, no differences emerged among groups, and mean pain scores remained substantially unchanged through day 90 (T90: PBM: 1.6 [0.96–2.27], MT: 0.9 [0.17–1.66], MT + PBM: 1.9 [0.79–2.92], all *p* < 0.05). The maximum opening analysis significantly improved between day 0 and day 90 in MT and MT + PBM groups (*p* = 0.023 and *p* = 0.05, respectively). Leite et al. [45] evaluated the effects of diacutaneous fibrolysis (DF) compared with sham treatment on myalgia and mouth opening in 34 women affected by TMD and myofascial pain. DF treatment was performed twice a week for approximately 10 min for a total of 4 weeks. Outcomes were assessed at T0 and T1 (4 weeks after the start of treatment). The experimental group showed an improvement in pain measured by VAS at the end of treatment (3.9 [IQR 0.7–8.2] vs. 0.6 [0–2.3]: *p* = 0.0001). Moreover, they showed a group difference at T1 (*p* = 0.02). The time‐by‐group interaction showed a statistically significant difference for the DF over time (*p* = 0.017) for mouth opening.

The trial by Serrano et al. [47] included 72 patients suffering from chronic myofascial TMD. In the experimental group, the patients received the pressure release technique treatment, whereas the control group underwent a sham treatment. Both groups were treated once a week for five weeks, with sessions lasting 45 min. VAS assessment was performed at T0, after treatment (T1), and at 3 months of follow‐up (T2). The experimental group showed a reduction in self‐reported pain compared to the control group at T1 and T2 (*p* < 0.001). Within the experimental group, pain decreased significantly from T0 to T1 (T0: 7.2 ± 0.9; T1: 4.9 ± 1.2; *p* < 0.001) and further at T2 (4.4 ± 1.8; *p* < 0.001). In the control group, a non‐clinically significant reduction in pain was observed at T1 and T2.

The experimental group reported significantly lower VAS pain scores compared to the sham group at both T1 and T2 (*p* < 0.001). Similarly, the sham group reported significantly lower VAS pain scores at T1 and T2 (*p* < 0.001), but the mean self‐reported VAS pain score was smaller than the a priori defined 1.2 cm clinically significant decrease.

Romeo et al. [48] included 62 patients with myogenic TMD. The participants were divided into two groups: the experimental group involving the combined use of occlusal splints, education, and musculoskeletal physiotherapy (including myofascial release treatments) and control group receiving only the occlusal splint and education. The experimental group underwent 10 physiotherapy sessions lasting 45 min each, spread over three months. Logistic regression analysis demonstrated that the experimental group had significantly higher probability to have a clinically important improvement by more than 3 times at VAS open (OR = 3.06, *p* = 0.04) and more than 6 times at VAS chewing (OR = 6.89, *p* < 0.01). In the study by Espejo Antunez et al. [49] 46 patients with diagnosis of myofascial pain were included. Patients in the experimental group underwent soft tissue mobilization and release techniques plus dynamic cervical electrical stimulation (electro‐massage), whereas patients in the control group underwent soft tissue mobilization and release techniques. Both therapies were delivered two sessions/week for two weeks and each session lasted for approximately 25 min. Significant time × group interactions were reported for self‐reported pain intensity (*p* < 0.001); and pressure pain sensitivity at the right masseter muscle (*p* < 0.001) and at the upper trapezius (right upper trapezius: *p* < 0.001; left upper trapezius: *p* < 0.001). Similarly, a significant time × group interaction was observed for vertical mouth opening (*p* < 0.001). Participants in the experimental group exhibited a greater improvement in pain related outcomes and mouth opening, with a large effect size.

In the trial conducted by Ferreira et al. [50], a total of 60 patients diagnosed with myogenic TMD were included. Subjects in the dry needling group underwent dry needling therapy, participants in the manual therapy group underwent a myofascial release treatment applied to the cervical, suboccipital, and temporomandibular areas, and subjects in the control group underwent cognitive behavioural therapy. Participants in the experimental groups received a 4‐week treatment, each session lasting 30 min. Outcomes were assessed at T0, post‐treatment, and 30 days after the last session. There was a decrease in VAS over time only in the dry needling and manual therapy groups (*p* < 0.001), and an interaction effect of time × group was reported after 30 days, with a difference for the dry needling and manual therapy groups (*p* = < 0.001).

The trial by Gurudut et al. [46] analysed 34 subjects with myofascial pain diagnosis. Subjects in the experimental group underwent soft tissue manipulation including massage and myofascial release for facial muscles for 5 sessions on alternate days, and subjects in the control group underwent drug therapy with chlorzoxazone 250 mg and aceclofenac 100 mg for 7 days twice daily. Both groups showed a statistically significant difference in pain (*p* = 0.001); however, no significant inter‐group difference was reported (*p* = 0.066) at the end of the treatment. When evaluating the between‐group differences in pain reduction against the pre‐specified MCID threshold of 15–22 mm, the clinical meaningfulness of the interventions varied across the included trials. In Brochado et al. [44], between‐group differences were not statistically significant, and within‐group reductions did not clearly surpass the MCID threshold.

The study by Espejo‐Antúnez et al. [50] showed a significant between‐group difference, with MCID threshold improved immediately after intervention (−1.59 cm) and at the 4‐week follow‐up (−1.90 cm).

Similarly, Romeo et al. (2024) [48] and Ferreira et al. [50] reported statistically significant *p*‐values for pain reduction, b indicating that the effects were clinically meaningful according to the established 15–22 mm criteria. Follow‐up assessment timelines varied across the included studies.

### 3.2 Evidence of Quality and Risk of Bias

**FIGURE 2:**
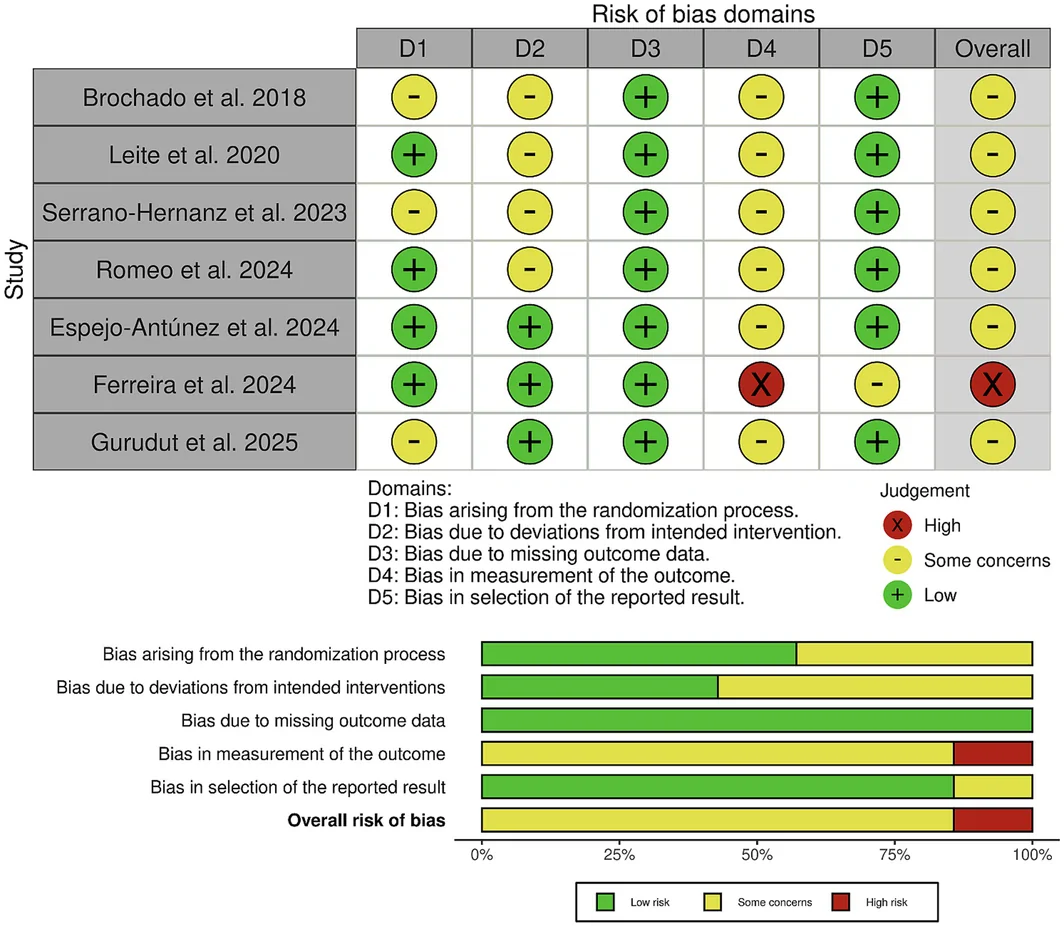
RoB 2 traffic‐light plot and ROB2 summary plot showing risk of bias across five domains for each included randomized trial. Colour coding indicates domain‐specific risk: Green, low risk; Yellow, some concerns; Red, high risk. An overall risk of bias per study is indicated by the colour in the corresponding row (per RoB 2 guidelines).

**FIGURE 3:**
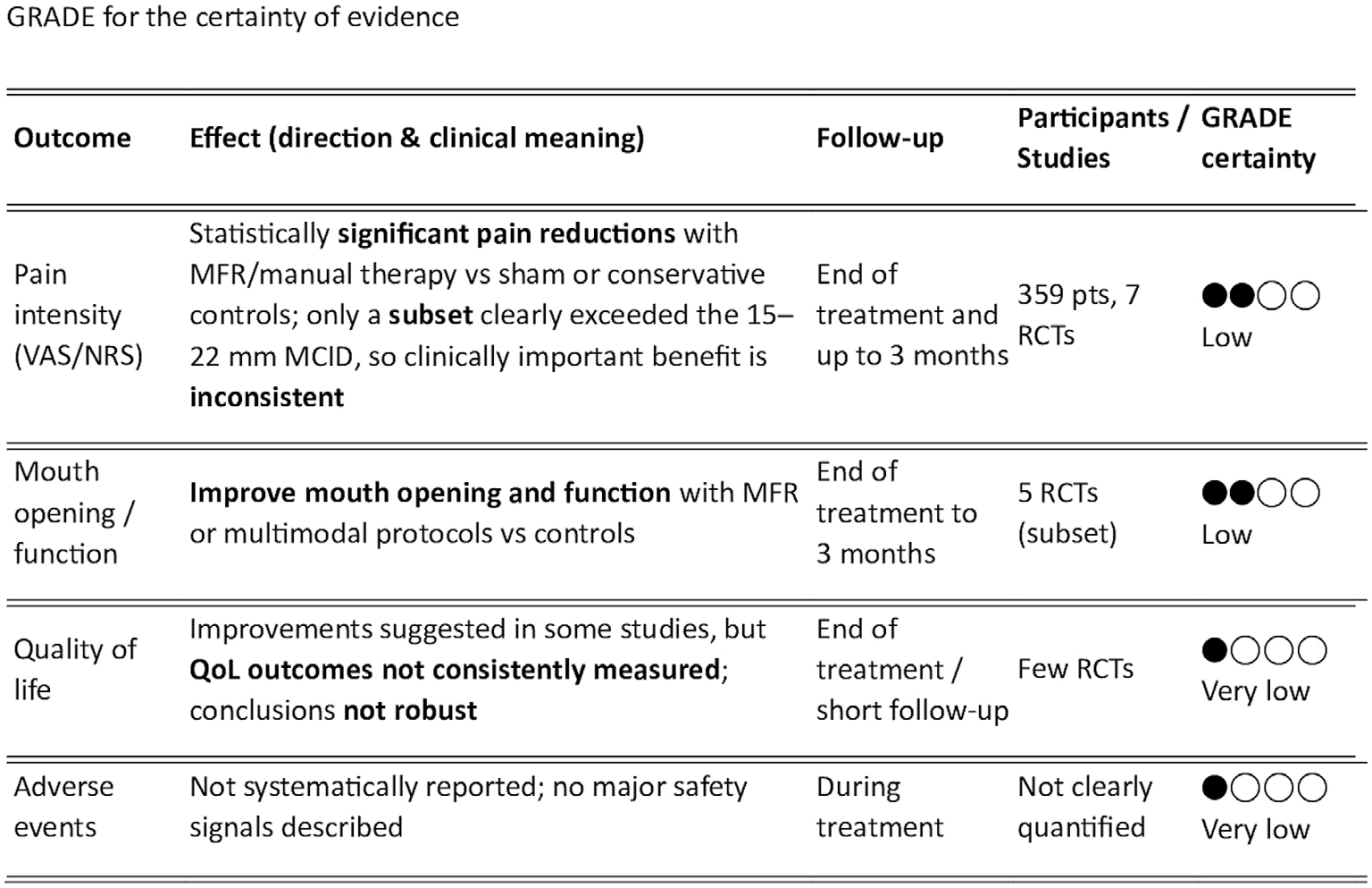
Summary of findings of pain reduction, mouth function, and QoL after MFR and soft tissue‐related techniques compared with baseline with GRADE certainty of a body of evidence.

The risk of bias of the included studies was assessed using the Cochrane RoB 2 tool (Figure 2). Inter‐rater agreement between the two independent assessors was strong, with a weighted Cohen's (\kappa) of 0.84. Overall, no study was judged to have a low risk of bias; six studies demonstrated “some concerns”, [44, 45, 46, 47, 48, 49] and one study [50] was judged to have a “high” risk of bias. Regarding Domain 1 (bias arising from the randomization process), three trials [44, 46, 47] were rated as having “some concerns.” This judgement was primarily due to an inadequate description of allocation concealment methods, rather than baseline imbalances, which were generally well‐controlled across the trials. For Domain 4 (bias in measurement of the outcome), judgements were heavily influenced by the nature of the interventions and outcomes. Because the primary outcome across trials was self‐reported pain (e.g., via Visual Analog Scale) and participants could not be blinded to the physical interventions (e.g., manual therapy versus sham or CBT), the RoB 2 algorithm dictates a minimum rating of “some concerns.” Consequently, all trials lacking participant blinding for self‐reported outcomes were rated as having “some concerns” in this domain. Ferreira et al. (2024) [50] was rated as “high risk” for Domain 4 because, in addition to the lack of blinding and use of self‐reported measures, the study context strongly suggested that the assessment of the outcome was likely influenced by the knowledge of the intervention received. Furthermore, the GRADE assessment showed a low certain of evidence for pain reduction and mouth function, and a very low certain of evidence in improving QoL (Figure 3).

## 4 Discussion

This study sought to gather scientific evidence on the effectiveness of MFR for the treatment of TMD‐myogenous related pain. For this aim, only controlled and randomized trials were included, and the included patients were standardized using the DC/TMD or the RDC/TMD, to ensure the validity, similarity, and reproducibility of the studies. The studies included several myofascial release techniques, including soft tissue mobilization, trigger point pressure in masticatory muscles, and stretch of soft tissue. Our synthesis suggests that myofascial release and related soft‐tissue techniques may reduce pain and improve mouth function in adults with TMD and, more specifically, myogenous TMD. Because included interventions are heterogeneous and frequently embedded within multimodal protocols, we caution against attributing effects to a single technique (e.g., MFR) in single use. The certainty of evidence varied across trials, and heterogeneity in intervention components, outcomes, and risk of bias warrants cautious interpretation. A critical finding of this systematic review is the distinction between statistical significance and clinical meaningfulness in the treatment of TMD pain. While several included trials reported statistically significant reductions in pain following manual therapy [46, 48, 49, 50], benchmarking these results against validated TMD‐specific MCID thresholds (15–22 mm) reveals a more nuanced clinical picture. Statistical significance alone is insufficient to guide patient‐centred care, as small, statistically detectable differences may not translate into perceptible relief for the patient. In our synthesis, only a subset of trials demonstrated between‐group pain reductions that confidently cleared the MCID threshold. This highlights a common gap in the current literature, where large, standardized effect sizes or low *p*‐values do not consistently guarantee substantial clinical benefit. Clinicians should interpret these findings cautiously, recognizing that while MFR is statistically superior to control interventions in several studies, the absolute magnitude of pain relief may not always meet the threshold for clinically meaningful improvement.

Pain represents one of the main symptoms TMD patients need treatment and assistance, and conservative, noninvasive, and reversible treatments are recommended as initial therapy for painful TMD [51]. Similar outcomes were underlined in a previous study where MFR techniques were assessed for their efficacy in TMD disorders [51, 52]. The effectiveness of MFR alone or in combination with others rehabilitative and not invasive treatment in patients with TMDs was assessed in this study. In this case, some researchers believe that myofascial release, especially in combination therapy, may be a suitable method for treating TMD [49]. Recent systematic review demonstrated that the combination of different physical therapies such as instrumental physical therapy, therapeutic exercise, manual therapy, spine manipulation, cognitive behavioural therapy, pain education, and physical activity improves pain and quality of life in TMD's patients [53, 54]. Regarding the long‐term effects of MFR in improving pain, our review shows that five studies have reported results in terms at follow‐up, with similar results in terms of improving pain at distance of the end of each treatment, until 3 months [44, 47, 48, 49, 50]. In the light of our systematic reviews, two RCTs have investigated the efficacy combination of MFR and the instrumental physical therapy, such as electrotherapy and photobiomodulation, demonstrating an improvement only with the combination of electrotherapy and MFR [44, 49]. Furthermore, it appears that multimodal and multidisciplinary therapeutic strategies may represent a valid therapeutic option for the TMD's patients. Interesting to note that only one study has compared MFR and analgesic oral drug therapy (chlorzoxazone 250 mg and aceclofenac 100 mg for 7 days twice daily), demonstrating no differences between MFR and oral analgesic drugs in improving pain and function [46].

Myofascial release is described as a general term for various manual treatment techniques with specific characteristics that exert pressure on muscles and myofascial tissue, which aims to relieve pain by restoring the function of damaged soft tissues [24, 30]. Myofascial tissue may be the source of pain in other musculoskeletal diseases, such as plantar fasciitis and chronic low back pain [55]. The theory of the therapeutic effect of MFR is based on the special role of fascia. Myofascial disease is the main factor determining musculoskeletal function and plays a vital role in the dynamic characteristics of muscle function [56]. On the other hand, MFR can reduce the pressure on pain‐sensitive structures such as nerves and blood vessels by improving the length and health of restricted connective tissues, reducing the inflammatory status affecting myofascia [46]. Although the specific mechanism of MFR is not yet clear, studies have found that MFR stimulates receptors distributed in the myofascial membrane, leading to neuromuscular changes [57]. Moreover, the central nervous system regulation of pain may be altered due to the occurrence of TMD [58]. It has been reported that most patients with TMD do not have intraarticular pathological changes but have chronic musculoskeletal dysfunction of the masticatory and cervical muscles and that treatment of these musculoskeletal disorders can effectively relieve the pain of patients [51]. At the same time, MFR may improve the patient's physical function by improving the patient's exercise status and jaw mobility [47]. Previous studies have shown that the pain threshold of patients with TMD is lower than that of healthy people, and the reduction of pain threshold is related to the decrease in the intensity of TMD and the reduction of physical function and nutritional support [59, 60]. In addition, the degree of pain and disability in patients with TMD may have a negative impact on the patient's quality of life (QoL), and, in this context, myofascial release may play a key role in improving QoL, exerting a positive role in pain relief and disability [61, 62]. At the same time, recent studies have found that psychosocial factors play a vital role in TMD [63, 64]. They have found that even in pain control, psychological factors still affect the temporomandibular mobility and cause abnormal muscle activity in patients with TMD [65, 66]. Nevertheless, quality of life outcomes were not consistently measured across trials; therefore, conclusions regarding QoL are not robust and should be interpreted with caution.

We are aware that this systematic review has some limitations. First, although the review was registered in PROSPERO, registration was not fully prospective with respect to the initial search process, as pilot work and formal study identification had already been completed and screening had started at the time of registration; second, the low number of included studies; third, there are concerns regarding the risk of bias in more than half of the evidence; fourth, the high heterogeneity in treatment modalities and in terms of follow‐up could have influenced the results; fifth, no cost‐effectiveness analysis was conducted in this systematic review; sixth, although the primary outcome of pain intensity was consistently measured using continuous scales (VAS or NRS), a quantitative meta‐analysis was not performed due to substantial clinical and methodological heterogeneity among the included trials. The studies varied widely in their comparator groups (ranging from sham interventions and standard pharmacological care to Cognitive Behavioural Therapy), specific manual therapy techniques applied, treatment frequencies, and follow‐up time points. Pooling data across such diverse clinical comparisons and control conditions can produce misleading pooled effect estimates and a qualitative synthesis was deemed the most robust approach. Lastly, the psychosocial disability was not evaluated through the Axis II DC/TMD questionnaires; thus, the investigation on the effects of different treatments according to a biopsychosocial model was not evaluated. Nevertheless, this systematic review synthesizes randomized controlled trials evaluating the effect of MFR on pain in adults with TMD. Although previous reviews have addressed MFR or manual therapy in broader rehabilitation contexts for TMD, those analyses often included non‐randomized designs or non‐specific interventions. Moreover, the review also updates the evidence base through trials published up to 2024–2026 and employs the Cochrane RoB 2 tool for risk of bias assessment [33, 53, 67].

## 5 Conclusion

MFR seemed to reduce pain and improve mouth function in patients with myogenous TMD. However, when benchmarked against established clinical thresholds, clinically meaningful efficacy (surpassing the 15–22 mm MCID) is only supported by a subset of the included trials. Pain reduction and improved function were observed in some trials, but certainty is low because of the risk of bias, heterogeneity, and modest, sometimes sub‐MCID effects. In this context, MFR could be taken into consideration for a multidisciplinary approach for TMD patients. More rigorous isolated‐MFR trials are needed to better characterize the efficacy of MFR in patients affected by myogenous TMD.

## Author Contributions

**Alfredo De Rosa:** writing – review and editing. **Dario Calafiore:** conceptualization, methodology, investigation, formal analysis, writing – original draft. **Antonio Ammendolia:** visualization. **Nicola Scotti:** writing – review and editing. **Martina Ferrillo:** conceptualization, methodology, investigation, formal analysis, writing – original draft, supervision. **Umile Giuseppe Longo:** visualization. **Alessandro de Sire:** conceptualization, methodology, investigation, formal analysis, writing – original draft, supervision. **Ambra Sedran:** writing – review and editing, writing – original draft. **Michele Vecchio:** visualization. **Amerigo Giudice:** writing – review and editing.

## Funding

The authors have nothing to report.

## Consent

The authors have nothing to report.

## Conflicts of Interest

The authors declare no conflicts of interest.

## Data Availability

The data that support the findings of this study are available from the corresponding author upon reasonable request.
